# Hyaluronic Acid/Trehalose Ophthalmic Solution in Reducing Post-Cataract Surgery Dry Eye Signs and Symptoms: A Prospective, Interventional, Randomized, Open-Label Study

**DOI:** 10.3390/jcm10204699

**Published:** 2021-10-13

**Authors:** Rita Mencucci, Eleonora Favuzza, Giulia Decandia, Michela Cennamo, Fabrizio Giansanti

**Affiliations:** Eye Clinic, Careggi Hospital, Department of Neuroscience, Psychology, Pharmacology and Child Health (NEUROFARBA), University of Florence, 50121 Florence, Italy; elefavuzza@gmail.com (E.F.); giuli_dec@hotmail.it (G.D.); michelacennamo@libero.it (M.C.); fabrizio.giansanti@unifi.it (F.G.)

**Keywords:** dry eye disease, cataract surgery, tear substitutes, artificial tears, trehalose, hyaluronic acid

## Abstract

The purpose of this prospective study was to evaluate the efficacy of the perioperative use of a hyaluronic acid (HA) and trehalose ophthalmic solution (Thealoz^®^ Duo) in reducing post-cataract surgery dry eye signs and symptoms in patients with mild/moderate dry eye disease (DED). One hundred and twenty patients, scheduled for unilateral cataract surgery, were randomized into three groups: (1) group A: HA/trehalose three times/day in the preoperative week and for 5 postoperative weeks; (2) group B: HA/trehalose for only 5 postoperative weeks; (3) group C: no artificial tears. In groups A and B, OSDI (Ocular Surface Disease Index) questionnaire scores were significantly lower than group C at all the postoperative visits; in group A they were significantly lower than group B on the day of surgery, with similar results in the first and fifth weeks after surgery. In groups A and B, break-up time (BUT) was significantly higher than group C during the postoperative period (*p* ≤ 0.001). In comparison to the preoperative values, BUT in group A remained stable 7 days after surgery; however, in groups B and C, it significantly decreased. In conclusion, the HA/trehalose ophthalmic solution effectively reduced post-cataract surgery DED signs and symptoms in patients with mild/moderate DED, particularly if also administered in the preoperative period.

## 1. Introduction

Despite being uneventful and uncomplicated, cataract surgery can cause dry eye symptoms and signs or worsen preoperative dry eye disease (DED), a condition which is often underdiagnosed [1].

The prevalence of DED in patients submitted for cataract surgery is indeed underestimated: this can be due to a discrepancy between signs and symptoms, and difficulties in formulating the diagnosis of DED [2,3,4]. Recently, an observational study by Trattler et al. [4] reported that, in their study cohort, 80% of patients had a tear break-up time (TBUT) <7 s and 50% showed corneal central staining; nevertheless, <25% of patients had been previously diagnosed with DED.

The incidence of DED after cataract surgery varies among studies, from 9.8 to 34% [5]. The onset, or the worsening, of DED signs and symptoms usually occurs in the first postoperative week and may last for months [3,6], affecting the satisfaction and quality of life of the patient.

In order to prevent onset or worsening DED after cataract surgery, the presence of DED, and its risk factors, should be investigated preoperatively [1]; optimizing the ocular surface before surgery is not only crucial in at-risk patients but can also be useful for all phacoemulsification candidates [7,8]. Moreover, appropriate intraoperative management—i.e., reducing ocular surface exposure to topical anesthetics and mydriatics, preservative-containing eye drops, to the light of the microscope and to the ultrasound [9]—and tailored postoperative treatment can effectively improve the quality of life of each surgical patient [1].

The mainstay of post-cataract surgery DED prevention and management is the treatment of Meibomian gland dysfunction (MGD) with non-preserved artificial tears, which has shown good results in terms of the reduction in postoperative DED signs and symptoms in previous clinical studies [1,7,8,10,11,12]. Thealoz^®^ Duo formulation (Laboratoires Thea, France) is the combination of a viscosity-enhancing agent, hyaluronic acid (HA) 0.15%, which improves lubrication and prolongs tear retention time on the ocular surface [13,14] and an osmoprotectant agent, trehalose 3%. Trehalose is a widely used ocular pharmaceutical agent, a natural disaccharide that has been shown to preserve the integrity of the cornea, conjunctival cells and their intracellular organelles through multiple mechanisms, including the control of inflammation, maintenance of homeostasis and protection against apoptosis [15,16,17]. Previous clinical reports have shown the efficacy of this combination in DED treatment [18,19,20,21]. In a recent study, a trehalose 3%/HA gel formulation effectively reduced the signs and symptoms of dry eye and improved tear film stability when administered after cataract surgery [11].

The purpose of our study was to evaluate the efficacy of the perioperative use of a hyaluronic acid (HA) 0.15% and trehalose 3% ophthalmic solution (Thealoz Duo), administered the week before cataract surgery and for 5 postoperative weeks, in reducing post-cataract surgery dry eye worsening in patients with mild/moderate dry eye disease (DED).

## 2. Materials and Methods

In this prospective, interventional, randomized, open-label study, 120 patients affected by mild/moderate DED who had been scheduled for unilateral cataract surgery were enrolled over a 5-month period (November 2020–March 2021). The study was conducted at the eye clinic of Careggi Hospital, University of Florence, Italy, according to the guidelines of the Declaration of Helsinki, and was approved by the Area Vasta Centro Ethics Committee on 20 February 2020 (code 16335_spe).

Patients were enrolled at the preoperative visit if they met all the following inclusion criteria: age > 55 years; patients scheduled for unilateral cataract surgery (phacoemulsification + IOL implantation); patients able and willing to provide voluntary written informed consent prior to any study-related procedure; patients with mild/moderate DED, with the following scores in the study eye at the preoperative visit (Visit 0):

- OSDI (Ocular Surface Disease Index) questionnaire score ≥13 and ≤32.
- Tear Film Break up Time (BUT) ≥5 and ≤10 s.
- Schirmer test I (without anesthesia) >5 mm and <15 mm.

Patients were excluded from the study if they met any of the following exclusion criteria: comorbidity with other severe or chronic ocular conditions that, in the judgment of the investigator, would have interfered with study assessment; use of topical glaucoma therapies or other concomitant ocular treatments; use of artificial tears within 30 days prior to Visit 0; severe meibomian gland dysfunction (MGD) assessed by slit-lamp examination; use of systemic drugs with anticholinergic activity (anticonvulsants, antihistamines, antipsychotics, antidepressants); changes to concomitant systemic therapies expected during the study; complicated cataract (post-traumatic, pseudoexfoliation syndrome, pharmacological mydriasis <6 mm, shallow anterior chamber based on investigator judgement); patients scheduled for femtosecond laser-assisted cataract surgery; cataract surgery procedures with corneal suture; corneal opacities or anterior corneal dystrophies; contact lens wearers; patients who were participating or had participated in other clinical studies 30 days prior to enrolment in the study.

Each patient underwent 4 visits: Visit 0, V0 (preoperative visit), between 30 and 5 days before surgery; V1, the day of surgery, before the administration of mydriatic agents; V2, 7 ± 3 days after surgery (one postoperative week); V3, 35 ± 3 days after surgery (5 postoperative weeks).

All patients followed the same antibiotic and anti-inflammatory postoperative topical regimen (chloramphenicol + betamethasone eye drops 4 times a day for 10 days after surgery, bromfenac eye drops twice a day for 30 days). At Visit 0, the patients were randomized into three groups according to a computer-generated randomization list in a 1:1:1 ratio:

(1) Group A: HA/trehalose ophthalmic solution 3 times/day in the preoperative week and for 5 postoperative weeks;
(2) Group B: HA/trehalose ophthalmic solution only for 5 postoperative weeks;
(3) Group C (control group): no additional artificial tears.

The following examinations were performed at all study visits: OSDI questionnaire [22], fluorescein BUT, corneal fluorescein staining and Schirmer test without anesthesia.

Corneal fluorescein staining (CFS) was evaluated using the Oxford grading scale, which divides corneal staining into 6 grades according to the severity, from 0 (absent) to 5 (severe) [23].

All surgeries were performed by the same experienced surgeon (R.M.); the surgical procedures involved a 2.2 mm sutureless temporal clear corneal incision and standard phacoemulsification with intraocular lens implantation.

### Statistical Analysis

The sample size was calculated assuming a clinically significant minimal score difference of 5 points in the OSDI questionnaire and a standard deviation of 13 points, using the OSDI score as a continuous variable [22,24]. We calculated the sample size to compare the average of a continuous variable between two groups. We adjusted this figure for multiple comparisons using the Bonferroni correction, setting the type I error to 0.017, a value obtained by dividing the conventional threshold by 3 (e.g., 0.05/3). The sample size for each group was 37 patients, which we increased to 40, considering a follow-up loss rate of less than 5% due to the short follow-up. We also calculated the sample size for a three-arm comparison using the one-way ANOVA test. Twenty-eight patients per group would be needed to detect a difference from the mean of +5, 0 and −5 in the three groups, given a standard deviation of 13. In conclusion, we chose a sample size of 40 patients per group following the previous calculation.

Collected data were analyzed and presented using descriptive statistics. In general, continuous variables were presented as a mean and standard deviation (SD), and categorical variables as numbers and percentages.

The following endpoints were evaluated in the efficacy analysis:

− Primary endpoint: to evaluate the efficacy of the HA/trehalose ophthalmic solution on dry eye symptoms when administered 7 days before surgery, compared to a group in which the solution was administered only after surgery and a group in which the solution was not administered (difference between the three groups in the score of the OSDI questionnaire at 5 postoperative weeks, V3).
− Secondary endpoints: differences in (BUT) score, corneal fluorescein staining score according to Oxford scale in the 3 groups at 5 postoperative weeks (V3).

The statistical analysis of continuous variables for the comparison of the three groups was carried out using the one-way ANOVA test combined with the Tukey post hoc test. The Student’s *t*-test for paired samples was used for intra-group comparisons. For categorical variables, Fisher exact test was used. The normal distribution of the variables was previously verified by the Shapiro–Wilk test of normality.

Statistical tests were conducted at a significance level of 0.05, and a 95% confidence interval was considered unless otherwise specified.

## 3. Results

This study initially included 123 eyes from 123 patients with a mean age of 73.71 years and a standard deviation of 8.98 years (minimum 55 years, maximum 88 years); 63.33% of the patients were women and 36.67% were men.

Group A consisted of 42 patients, group B of 41 patients and group C of 40 patients.

Three patients (two in group A and one in group B) decided to leave the study for personal reasons between Visit 0 and Visit 1 (before cataract surgery). These patients were subsequently excluded from the analysis, which then left 40 patients per group. No patient was lost to follow-up.

In Table 1, the scores of the OSDI questionnaire, BUT, Schirmer test and fluorescein staining (corneal staining, according to the Oxford classification) at Visit 0 are reported. There was no significant difference between groups (*p* > 0.05, ANOVA test).

### 3.1. OSDI Score

**Figure 1:**
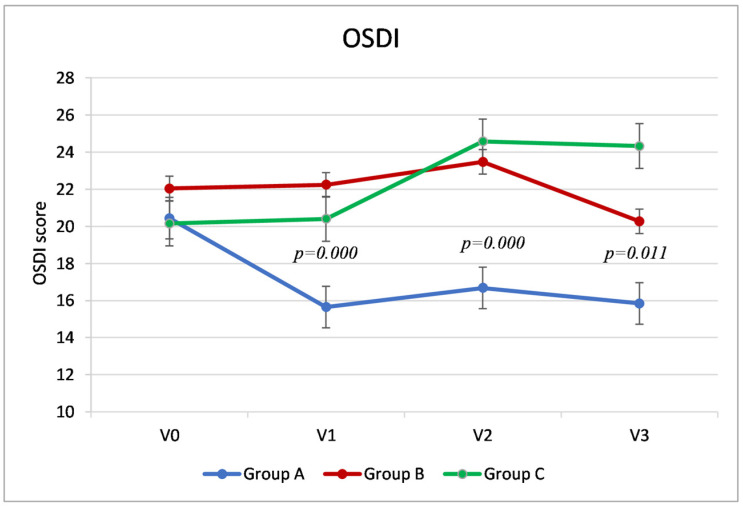
OSDI (Ocular Surface Disease Index) questionnaire scores. The reported *p*-values refer to the comparisons between Groups A and B at V1, V2 and V3.

The primary outcome of the study, the score of the OSDI questionnaire at the final visit (V3, at 5 weeks after cataract surgery), was significantly different between the three groups: in particular, Group A reported a score 4.42 points lower (fewer symptoms) than group B (95% confidence interval, CI, −8.01–−0.83, *p* = 0.011) and 8.48 points lower than group C (CI95 % −12.07–−4.89, *p* = 0.000). Group B showed a score 4.06 points lower than group C (95% CI −8.23–−1.27, *p* = 0.023). (Table 2, Figure 1).

Group A showed significantly lower OSDI values (fewer symptoms) than the other two groups during V1 and V2. Specifically, at Visit 1, Group A showed an average difference compared to Group B of 6.58 points (95% CI −10.06–−3.11, *p* = 0.000) and 4.75 compared to Group C (95% CI −8.23–−1.27, *p* = 0.004); at Visit 2, Group A had a score 6.79 points lower than Group B (−10.67–−2.91, *p* = 0.000) and 7.89 points lower than Group C (95% CI, −11.77–−4.01, *p* = 0.000). Conversely, during Visits 1 and 2, the scores of Groups B and C were similar (*p* > 0.05). (Table 2, Figure 1).

Analyzing the trend of the OSDI score within the three groups (Figure 1), in Groups B and C, the values remained stable between the preoperative visit (V0) and the day of surgery (V1) (as was expected since they had not instilled any artificial tears into the operated eye). In Group A, the group that instilled the HA/trehalose ophthalmic solution from 7 days before the cataract surgery, there was an average reduction in symptoms of about 5 points (4.79), which was statistically significant (*p* = 0.000, *t*-test for paired samples). At Visit 2, 7 days after surgery, there was a significant increase in OSDI compared to the preoperative visit, which indicated a worsening of symptoms in Groups B and C (respectively, *p* = 0.003 and *p* = 0.000), but only clinically significant in the latter group (a mean increase of about 4 points). In Group A, on the other hand, the OSDI score remained stable even after cataract surgery. At Visit 3, 5 weeks after surgery, while Group A and Group B reported OSDI values similar to V0 (in Group A, even lower), Group C instead reported greater clinical symptoms than during preoperative visits (a mean difference of about four points between V3 and V0, *p* = 0.010, *t*-test for paired samples).

### 3.2. BUT

**Figure 2:**
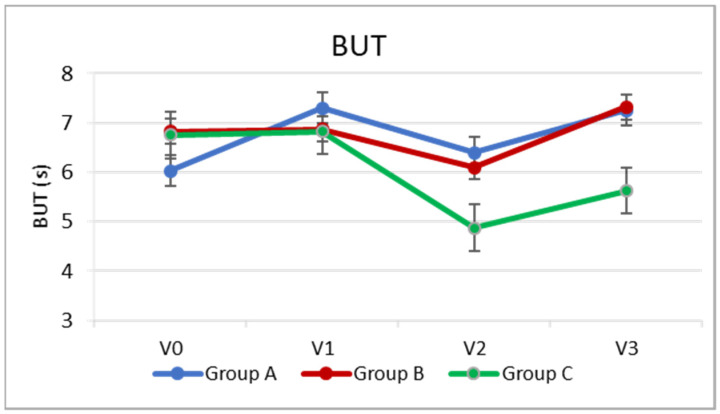
Fluorescein break-up time (BUT) at each study visit.

Regarding the secondary outcomes, at the two postoperative visits (V2 and V3), BUT was significantly higher in Groups A and B than in Group C, with no significant difference between Groups A and B (*p* = 0.001 group B vs. C, *p* = 0.000 A vs. C, *p* > 0.05 group A vs. B). (Table 2, Figure 2).

In Group A, there was a slight increase, or slight improvement, in BUT on the day of cataract surgery (V1), approximately 1.3 s (*p* = 0.000), compared to the preoperative visit (V0). The BUT then remained relatively stable even following cataract surgery (*p* > 0.05), and at V3, it was slightly higher than the preoperative level (*p* = 0.000) by about one point. In Group B, there was a slight but significant reduction of about one point (*p* = 0.004) 7 days after cataract surgery compared to baseline, while at V3, the BUT returned similar to the baseline (*p* > 0.05). In the control group, which did not instill any artificial tears into the operated eye, the reduction in BUT after cataract surgery was greater (by about two points, *p* = 0.000), and 5 weeks after surgery, the BUT value remained approximately one point lower than at V0 (*p* = 0.003). (Table 2, Figure 2).

### 3.3. Corneal Fluorescein Staining

**Figure 3:**
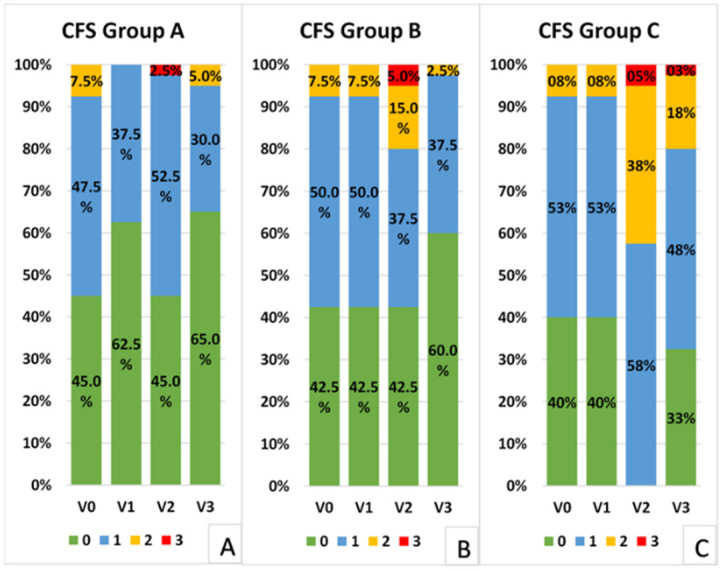
Corneal fluorescein staining (CFS, Oxford scale) distribution in the three groups: (**A**) Group A; (**B**) Group B; (**C**) Group C.

The distribution of corneal fluorescein staining scores (Oxford Scale) was significantly different in Groups A and B compared to Group C at all the postoperative visits. In fact, a greater number of patients showed staining greater than, or equal to, one in the control group compared to the other two groups at Visits 2 (*p* = 0.000, Fisher test) and 3 (*p* = 0.007 Group A vs. C, *p* = 0.024 Group B vs. C) (Figure 3). Groups A and B presented a similar distribution of scores at all visits (*p* > 0.05, Fisher test).

## 4. Discussion

Cataract surgery is one of the most commonly performed ocular procedures, with excellent visual outcomes [5]. Nevertheless, it is one of the main causes of iatrogenic dry eye disease, especially in patients with risk factors such as old age, female sex, preoperative ocular surface disease or DED and contact lens use [1,4,5]. Post-cataract surgery DED is often a transient condition lasting up to 1–3 months, but it can become chronic in 10% of cases, affecting the patients’ quality of life [2,3,25].

Several treatments are available for DED, and postoperative management should address and modulate the factors that contribute to the vicious cycle of DED and to loss of ocular surface homeostasis (inflammation, epithelial dysfunction, tear instability, nerve impairment and MGD), according to the severity of the disease [26]. Artificial tears are the first choice of therapy for all forms of DED [13]. The association between viscosity-enhancing agents such as hyaluronic acid and osmoprotectant agents such as trehalose has shown more beneficial effects after cataract surgery than hyaluronic acid alone [12]. Moreover, the gel formulation of hyaluronic acid/trehalose has been reported to reduce the signs and symptoms of DED and improve tear film stability compared to 0.9% unpreserved sodium chloride eye drops when administered for 4 weeks after cataract surgery [11].

Nevertheless, the presence of DED and its risk factors should be investigated preoperatively to avoid postoperative complications [1], thereby optimizing the ocular surface before surgery. A previous study of eyes not affected by preoperative DED [8] showed that a hydroxypropyl guar and the hyaluronic acid ophthalmic solution was effective in reducing post-cataract surgery ocular discomfort and tear instability, with higher BUT and lower symptoms when also administered in the preoperative period. In another multicentric study, the use of a lubricating solution two weeks before cataract surgery, in eyes with either preoperative healthy ocular surface or mild DED [7], had a protective effect against postoperative DED.

In our study regarding patients affected by mild/moderate preoperative DED who did not instill artificial tears regularly, the group treated with HA/trehalose eye drops starting from the preoperative weeks (Group A) showed fewer dry eye symptoms (lower OSDI score) on the day of surgery and at the postoperative visits than the group treated only after surgery and the control group. Moreover, only in the group treated preoperatively did the OSDI score remain stable after cataract surgery compared to the preoperative visit. At 5 weeks after surgery, only the two groups treated with the ophthalmic solution reported OSDI scores similar to the preoperative visit, while in the control group, the score remained worse.

Among the signs of DED, BUT, at the two postoperative visits, was significantly higher in the two groups treated with artificial tears (groups A and B) compared to the control group, without significant difference between groups A and B. Nevertheless, only in the group treated preoperatively, was BUT higher on the day of surgery and remained stable after cataract surgery. Conversely, in the group treated only postoperatively, there was a slight reduction 7 days after cataract surgery compared to baseline, while at 5 postoperative weeks, the BUT returned similar to baseline. In the control group, which did not instill any artificial tears into the operated eye, the reduction in BUT after cataract surgery was greater and the BUT value remained slightly lower than baseline at the last visit.

Regarding corneal fluorescein staining, in the control group a greater number of patients showed staining higher than, or equal to, one compared to the other two groups at the postoperative visits.

Therefore, in our study, the preoperative administration of a HA/trehalose ophthalmic solution showed a protective effect, as it was associated with higher BUT and fewer DED symptoms on the day of surgery compared to the other groups; moreover, the group treated preoperatively showed greater stability of DED signs and symptoms after cataract surgery. Our results confirm that a better ocular surface status on the day of surgery can reduce the risk of postoperative ocular surface impairment.

The main limitation of this study was its short follow-up period. Further studies should also analyze the long-term effect of this perioperative treatment.

In conclusion, the hyaluronic acid 0.15% and trehalose 3% ophthalmic solution was effective in reducing post-cataract surgery dry eye signs and symptoms in patients with preoperative mild/moderate DED, particularly when the solution was also administered in the preoperative period.

The presence of DED and its risk factors should be investigated before cataract surgery in order to optimize the ocular surface, thus improving the postoperative outcome and avoiding ocular surface complications.

## Figures and Tables

**Table 1:** Preoperative data of enrolled patients.

	Group A	Group B	Group C	*p*-Value
**Age (years)**	73.80 ± 9.18	72.60 ± 10.13	74.73 ± 7.55	0.574 ^a^
**OSDI**	20.44 ± 7.33	22.04 ± 6.70	20.15 ± 7.00	0.431 ^a^
**BUT (s)**	6.03 ± 1.46	6.83 ± 1.72	6.75 ± 1.75	0.061 ^a^
**Schirmer test I (mm)**	10.45 ± 5.64	11.05 ± 6.40	12.43 ± 6.41	0.343 ^a^
**CFS score n (%)**				0.997 ^b^
**0**	18 (45%)	17 (42.5%)	16 (40%)	
**1**	19 (47.5%)	20 (50%)	21 (52.5%)	
**2**	3 (7.5%)	3 (7.5%)	3 (7.5%)	

Age, Schirmer test I, OSDI score and BUT are reported as mean ± SD. ^a^ ANOVA test; ^b^ Fisher exact test. Abbreviations: OSDI, Ocular Surface Disease Index; BUT, break-up time; CFS, corneal fluorescein staining; SD, standard deviation.

**Table 2:** OSDI score, BUT and Schirmer test I results in the three groups.

	Group A	Group B	Group C	*p*-Value (ANOVA)	*p*-Value (Tukey Post Hoc Test)		
					A vs. B	A vs. C	B vs. C
**OSDI score**							
**V0**	20.44 ± 7.24	22.04 ± 6.70	20.15 ± 6.70	0.431			
**V1**	15.65 ± 6.08	22.23 ± 6.45	20.40 ± 7.08	**0.000**	**0.000**	**0.004**	0.425
**V2**	16.68 ± 6.66	23.47 ± 6.30	24.57 ± 8.75	**0.000**	**0.000**	**0.000**	0.780
**V3**	15.84 ± 7.34	20.27 ± 5.40	24.32 ± 7.36	**0.000**	**0.011**	**0.000**	**0.023**
**BUT (s)**							
**V0**	6.03 ± 1.46	6.83 ± 1.72	6.75 ± 1.75	0.061			
**V1**	7.30 ± 1.38	6.88 ± 1.62	6.83 ± 1.52	0.306			
**V2**	6.40 ± 1.41	6.10 ± 1.75	4.88 ± 1.14	**0.000**	0.627	**0.000**	**0.001**
**V3**	7.25 ± 1.31	7.33 ± 1.38	5.63 ± 1.61	**0.000**	**0.971**	**0.000**	**0.000**
**Schirmer test I (mm)**							
**V0**	10.45 ± 5.64	11.05 ± 6.40	12.43 ± 6.42	0.343			
**V1**	10.55 ± 4.87	10.93 ± 6.13	11.95 ± 5.69	0.512			
**V2**	10.80 ± 6.70	11.13 ± 8.05	11.82 ± 7.35	0.818			
**V3**	12.22 ± 6.49	12.68 ± 7.97	12.12 ± 8.70	0.944			

Results are reported as mean ± SD. *p*-values highlighted in bold are statistically significant. Abbreviations: OSDI, Ocular Surface Disease Index; BUT, break-up time; SD, standard deviation; s, seconds; ANOVA, analysis of variance; vs., versus; V0, preoperative visit; V1, the day of surgery; V2, one postoperative week; V3, 5 postoperative weeks.

## Data Availability

Data available on request due to privacy/ethical restrictions.
